# Effect of Modified Red Pottery Clay on the Moisture Absorption Behavior and Weatherability of Polyethylene-Based Wood-Plastic Composites

**DOI:** 10.3390/ma10020111

**Published:** 2017-01-26

**Authors:** Qingde Li, Xun Gao, Wanli Cheng, Guangping Han

**Affiliations:** 1 Key Laboratory of Bio-based Material Science and Technology (Ministry of Education), Northeast Forestry University, Harbin 150040, China; liqingde2017@hotmail.com (Q.L.); gaoxunbeihuauniversity@hotmail.com (X.G.); guangping.han@nefu.edu.cn (G.H.); 2 College of Arts and Design, Qiqihar University, Qiqihar 161000, China

**Keywords:** silane coupling agent, red pottery clay, chemical modification, thermo-gravimetric analysis, UV-accelerated aging

## Abstract

Red pottery clay (RPC) was modified using a silane coupling agent, and the modified RPC (mRPC) was then used to enhance the performance of high-density polyethylene-based wood-plastic composites. The effect of the mRPC content on the performances of the composites was investigated through Fourier transform infrared spectrometry, differential mechanical analysis (DMA) and ultraviolet (UV)-accelerated aging tests. After adding the mRPC, a moisture adsorption hysteresis was observed. The DMA results indicated that the mRPC effectively enhanced the rigidity and elasticity of the composites. The mRPC affected the thermal gravimetric, leading to a reduction of the thermal degradation rate and a right-shift of the thermal degradation peak; the initial thermal degradation temperature was increased. After 3000 h of UV-accelerated aging, the flexural strength and impact strength both declined. For aging time between 0 and 1000 h, the increase in amplitude of ΔL* (luminescence) and ΔE* (color) reached a maximum; the surface fading did not became obvious. ΔL* and ΔE* increased more significantly between 1000 and 2000 h. These characterization results indicate that the chromophores of the mRPC became briefly active. However, when the aging times were higher than 2000 h, the photo-degradation reaction was effectively prevented by adding the mRPC. The best overall enhancement was observed for an mRPC mass percentage of 5%, with a storage modulus of 3264 MPa and an increase in loss modulus by 16.8%, the best anti-aging performance and the lowest degree of color fading.

## 1. Introduction

Biomass composites degrade readily when they are exposed to extreme natural environmental conditions. In such an environment, the durability and structure of the matrix will change [1,2]; photodegradation will occur [3,4]. For instance, exposure to ultraviolet (UV) light ultimately decreases the moisture absorption characteristics, the weatherability and the thermal properties of composites, a process referred to as UV-accelerated aging [5,6]. Fabiyi et al. studied the weatherability of wood powder/PVC composites. After an UV-accelerated aging process, the number of surface hydroxyl groups was found to increase, the color faded and the brightness increased, whereas the UV-accelerated aging performance of the composites did not show an enhancement [7]. Therefore, it is important to improve the properties of composite materials to increase their resistance to UV-accelerated aging.

Several studies have been conducted to improve the moisture adsorption and weatherability of biomass composites. Liu et al. studied the moisture absorption characteristics of south yellow pine/high-density polyethylene (HDPE) composites. They compared the moisture absorption and desorption behavior with the Nelson adsorption isothermal curve obtained for wood-plastic composites (WPCs) samples under the same temperature and humidity conditions and established a prediction model [8]. Deka et al. blended high-density polyethylene, wood powder and nanoclay to prepare nano-composite materials. They reported that, when adding 3 wt % of organic montmorillonite, the clay showed a better dispersion, a better thermal stability, an enhanced energy storage modulus and a higher loss modulus. However, the moisture adsorption properties decreased, and the weatherability became worse [9]. Kusuktham et al. introduced calcium silicate as a strengthening phase into WPCs, which improved the weatherability, but led to a certain irritant of the material [10].

So far, scholars have been conducting work to improve the thermal stability, the dynamic thermal-mechanical properties and the mechanical properties of biomass composites. Sanchez-Valdes et al. used a twin double screw extruder to process a polyethylene melt and used ammonium salt surfactants to modify the montmorillonite mixture, resulting in a higher viscosity of the fabricated nano-composite materials. The higher clay content led to a poorer dispersion or intercalation effect. The thermal stability of the composites were improved [11]. Kayaisang et al. used RPET (recycled polyester) as a substitute for LCP (liquid crystalline polyester) and then prepared thermoplastic fiber composites with an enhanced strength and a better thermal stability. However, the effect of the substitution on the mechanical properties has not been further studied [12]. Ramachandran et al. [13] and Anjana et al. [14] performed a surface analysis to optimize the processing technology for the fabrication of PP/HDPE/nano-kaolinite clay composites. Their results indicated that the thermal stability and dynamic thermal-mechanical properties of the composites were improved through the optimization of the processing parameters. However, an in-depth study on the effect of this optimization on the weatherability has yet to be conducted. Sibeko et al. studied the effect of a vinyltriethoxysilane treatment and the nanoclay content on the properties of low-density polyethylene/nanoclay composites and reported that their thermal stability remained almost constant [15]. Mohan et al. applied porous silica foam to enhance high-density polyethylene composites that effectively improved the thermal properties and the mechanical properties. However, it also resulted in certain brittleness [16]. Roumeli et al. studied the effect of maleic anhydride on the properties of bast fiber/high-density polyethylene composites. Their results showed that thermal properties and the mechanical of the composites both significantly improved, but the production costs increased, as well [17]. However, the dynamic mechanical properties and the stretching properties of the low-density polyethylene changed due to the presence of the vinyltriethoxysilane and the nanoclay. Elshereafy et al. added organic montmorillonite as a filler material to their ethylene propylene rubber/styrene-butadiene rubber/discarded high-density polyethylene composites, thereby improving their mechanical properties and thermal stability and increasing the gamma radiation dose [18].

Red pottery clay (RPC) is abundant and inexpensive. Its preparation process is simple and environmental-friendly. Adding RPC to biomass composites might effectively reduce the production costs and enhance the mechanical properties and weatherability of composites. This study explored the modification of RPC with silane coupling agents and used the modified RPC (mRPC) as a filler to reinforce HDPE/wood fiber composites. Furthermore, we investigated the effect of the mRPC content on the moisture adsorption characteristics, the UV-accelerated aging performance, the mechanical properties, the chroma, the dynamic mechanical thermal properties and the thermal gravimetric of WPCs. The results are expected to provide a theoretical foundation for applying RPC as a reinforcing agent and filler. Meanwhile, it provides a new method to enhance the properties of HDPE/wood fiber composites.

## 2. Experimental Section

### 2.1. Materials

Wood fibers with 40–60 mesh pulverized from poplar residue was placed into a 103 ± 2 °C electric oven (DHG-9140 type, Yiheng Experimental Instrument Co., Ltd., Shanghai, China) and dried to a moisture content lower than 3%. High-density polyethylene (HDPE) with a density of 950 kg/m^3^ and a melt flow index of 0.5 g/10 min (Petrochemical Co., Ltd., Daqing, China) was used as received.

The red pottery clay (RPC) was prepared according to the proportions listed in Table 1 and was soaked in water in a bucket for 24 h. Then, the mixture was placed in a TC-65 vacuum pug mill (Hebi Machinery Factory, Hebi, China) for refining. The refining procedure was repeated eight times in order to achieve the required tenacity. Subsequently, RPC was soaked in water for better plasticity. Finally, the refined RPC was dried to a moisture content of below 3%, ground and screened using an 80 mesh sieve.

The silane coupling agent (KH-550: NH_2_CH_2_CH_2_CH_2_Si(OC_2_H_5_)_3_) with a boiling point of 217 °C (Quanxi Chemical Co., Ltd., Nanjing, China), industrial-grade paraffin, MgCl_2_, NaNO_2_, NaCl, KaCl and Na_2_SO_4_ were used as raw materials in this study.

### 2.2. The Preparation of Samples

The silane coupling agent was mixed with the RPC at a mass ratio of 4:100 in a high-speed mixer (SHR-10A, Tonghe Plastic Machinery Co., Ltd., Zhangjiagang, China) at a temperature of 60 °C. Afterwards, the sample was dried at 80 °C for 6 h, and the modified RPC (mRPC) was obtained. The wood fibers, high density polyethylene (HDPE), maleic anhydride grafted polyethylene (MAPE) and lubricants (paraffin) were mixed according to the ratios of 38%, 58%, 3% and 1%, respectively. Then, the five mixtures were separately prepared, and the mRPC were added into the preceding mixture according to the ratios of 0%, 3%, 5%, 7% and 10% (the amount of mRPC is a percentage of the total amount of HDPE, wood fiber, MAPE and lubricants). The five mixtures were fed into a high-speed mixer for 15 min. The sample was extruded and molded using a twin-screw plastic extruder (Type SJSH30/SJ45, Nanjing Rubber Machinery Plant, Nanjing, China). The standard samples were prepared using electric saws and instruments for the preparation of the dumbbell-shaped sample.

### 2.3. Fourier Transform Infrared Spectroscopy

Red pottery clay (RPC) was modified using a silane coupling agent and then pressed into pellets. The functional groups on the surface were studied by Fourier transform infrared spectroscopy (FTIR) using a Nicolet 6700 FTIR spectrometer (Thermo Fisher Scientific Co., Ltd., Waltham, MA, USA) with a scanning range of 3500–400 cm^−1^, a resolution of 4 cm^–1^ and a scanning rate of 32 scans/min [19].

### 2.4. Sorption Measurements

Sixteen specimens with a size of 10 mm × 10 mm × 4 mm from each of the material types were randomly selected and numbered. After conditioning, eight specimens were oven-dried at 103 °C to reach the dry state for adsorption test. The remaining eight specimens were conditioned over distilled water to reach the fiber saturation state for the desorption test. All of the samples were conditioned to reach equilibrium at a relative humidity (RH) of 33%, 66% and 76%, 75%, 85% and 93%, respectively, over different saturated salt solutions in desiccators. The initial weight of all specimens was measured. The equilibrium moisture content (EMC) of each specimen was calculated based on the oven-dry weight.

### 2.5. Dynamic Mechanical Analysis Test

A dynamic mechanical analysis (DMA) can be used to investigate the dynamic thermomechanical properties of specimens through measuring and analyzing the specimen’s feedback to an acting force. The dynamic viscoelasticity of the prepared specimens was analyzed using a Q800 dynamic mechanical analyzer (TA Instruments, New Castle, DE, USA). The measurements were performed from −40 to 130 °C at a constant frequency of 1 Hz and a heating rate of 3 °C/min [20]. Three replicates with dimensions of 35 mm × 12 mm × 3 mm were tested for each type of composite.

The interfacial bonding between wood fiber and polymer matrix can be evaluated using the adhesion factor (*A*), which is determined from DMA data at 40 °C based on the study of Kubat et al. [21] by using Equation (1):

(1) $$A=\left(1/(1-Vf)\right)(\tan\delta_{c}/\tan\delta_{m})-1$$

where *c* and *m* subscripts denote composite and matrix, respectively, and *V_f_* is the fiber volume fraction. The low value of *A* is an indicator of good adhesion or a high degree of interaction between the two phases and vice versa.

### 2.6. Thermogravimetry Analysis Test

Thermogravimetry analysis (TGA) was carried out using a 309F3TGA analyzer (TA Instruments, New Castle, DE, USA). An approximately 5 mg sample was placed in an aluminum pan and heated at a constant heating rate of 10 °C/min under a nitrogen atmosphere. The final temperature inside the furnace was maintained at 600 °C (held for 2 min), and the carrier gas (argon) flow rate was kept at 50 mL/min. The mass loss curves and their derivatives were collected and calculated accordingly [22].

### 2.7. UV-Accelerated Aging Test

For the aging test, the sample was placed into a Q-panel UV aging tester (QUV/SPRAY, Q-Panel Company, Cleveland, OH, USA). According to the aging program based on the ASTMG-154 standard, the aging process uses a period of 12 h and is divided into two stages. During the first stage, the tester chamber was equipped with UVA-340 fluorescent bulbs to produce an accurate simulation of sunlight in the critical short wavelength region of the spectrum; wavelengths from the solar cut-off of 295–365 nm at an irradiation of 0.89 W/m^2^. The chamber was operated under repeated aging cycle conditions of 8 h UV exposure at 60 °C in a dry environment [23]. During the second stage, the samples with size of 76.2 mm × 76.2 mm × 3 mm were placed in the chamber for aging at 50 °C without UV radiation [24]. After aging for 500, l000, 1500, 2000, 2500 and 3000 h, the performances of the samples were tested and the mean value of five samples was used as the test result.

### 2.8. Colorimetric Test

The procedure outlined in ASTM D2244 served as the means by which to determine the color measurements on the surfaces of coextruded and control samples. A Minolta CR-420 Chroma Meter (Konica Minolta Corp., Fairfield, NJ, USA) measured the color in L*a*b* coordinates at three locations on each sample using the CIELAB system developed by the International Commission on Illumination (CUE, 1976) [25]. In this system, the L* axis (+L* for light, −L* for dark) represents the lightness and a* (+a* is for red, −a* for green) and b* (+b* for yellow and −b* for blue) the chromaticity coordinates. At least four replicates were measured for each formulation to obtain the average values of color. Calculations incorporated the values of lightness and chromaticity coordinates before and after weathering tests to determine the discoloration (ΔE*) of the weathered samples using the following equation [26,27]:

ΔE* = (ΔL*^2^ + Δa*^2^ + Δb*^2^)^1/2^ (2)

ΔL* = L* − *L*_0_* (3)

Δa* = a* − *a*_0_* (4)

Δb* = b* − *b*_0_* (5)

with ΔL*, Δa* and Δb* as the difference of initial and final values of L*, a* and b*.

### 2.9. Mechanical Properties Test

The mechanical properties of the prepared mRPC/HDPE/wood fiber composites samples were measured before and after the UV-accelerated aging. Samples for flexural and impact tests were prepared with sizes of 80 mm × 13 mm × 4 mm and 80 mm × 10 mm × 4 mm, respectively. The specimens for flexural testing were measured under three-point bending using an RGT-20A electronic mechanics testing machine (Shenzhen REGER Instruments Co., Ltd., Shenzhen, China) in accordance with ASTM D790 [28]. A crosshead speed of 2 mm/min and a span length of 64 mm were used for the test. Impact strength was determined using an HTS-CJY7780B impact tester (Zhongye Instrument Equipment Co., Ltd., Dongguan, China) according to ASTM D5628 [29]. Five replicate specimens were taken for each test, and average data along with corresponding standard deviation were reported.

### 2.10. Scanning Electron Microscopy

The surface morphology of the composite after the UV-accelerated aging was examined by a QUANTA 200 electron microscope (FEI Company, Eindhoven, The Netherlands) at ×1000 magnification. To study the micro-morphology, the specimens were completely frozen in liquid nitrogen to impede the plastic deformation of the matrix and to obtain a well-defined fiber-matrix interface. A cross-section with a thickness lower than 3 mm was selected for SEM investigations. The samples were coated with platinum prior to the observation to improve the surface conductivity and observed at an acceleration voltage of 15 kV. The samples were mounted on the aluminum sample holder and placed in the specimen chamber in a vacuum condition of 0.06 mbar at room temperature.

## 3. Results and Discussion

### 3.1. FTIR Analysis

**Figure 1:**
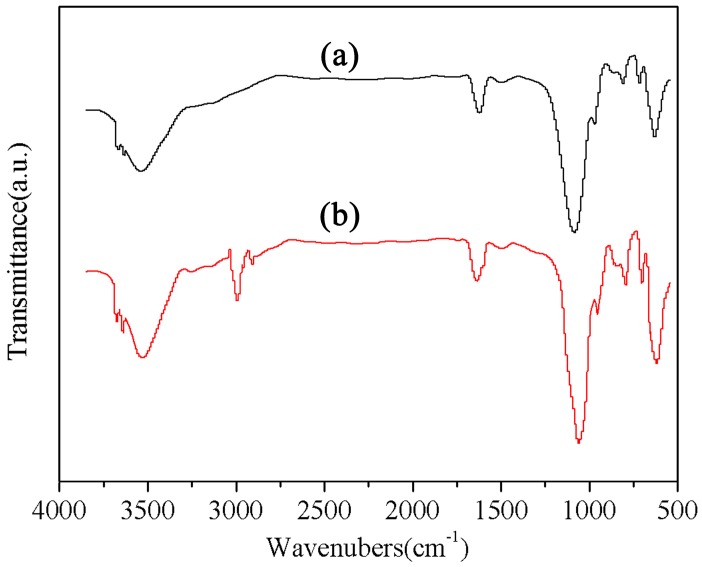
FTIR spectra of (a) unmodified red pottery clay (RPC) and (b) modified RPC (mRPC).

Compared with the control (unmodified RPC), the FTIR spectrum of mRPC did not change significantly. As shown in Figure 1, the spectrum can be divided into three regions according to the predominant features, i.e., the water vibration at high wave numbers, the Si–O stretching vibrations in the scope from 1200–800 cm^−1^ and the Al–O and Si–O flexural vibrations at wave numbers below 800 cm^−1^. New adsorption peaks appeared at 2837.84 and 2942.17 cm^−1^; these phenomena are attributed to the symmetrical stretching vibration of –CH_2_ and the characteristic adsorption of –CH_3_, under the action of the high temperature, each pair of free hydroxyl groups of the cellulose chains was subject to a “bridging reaction”, this resulting in the formation of ether bonds [30]. The Si–O–H group of the hydrolyzed silane coupling agent can form a hydrogen bond with a hydroxyl group on the surface of the RPC, thereby decreasing the number of free hydroxyl groups, which indicates that the coupling agent has been adsorbed on the surface of the RPC particles. Meanwhile, the silanol groups generated by the hydrolyzed silane can form covalent Si–O–C bonds with the RPC. The presence of both bonds indicates that the coupling agent was grafted onto the RPC via a co-polymerization reaction. This chemical reaction affected the surface composition of the RPC particles to some extent.

### 3.2. Effect of the Mass Percentage of Added mRPC on the Moisture Adsorption Characteristics of the Composites

**Figure 2:**
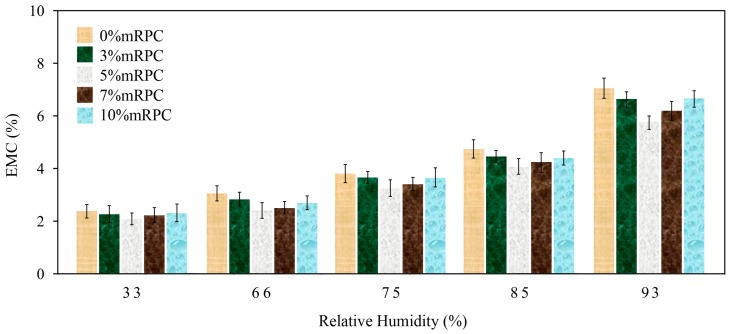
Comparison of the EMCs of the composites along the moisture adsorption direction.

**Figure 3:**
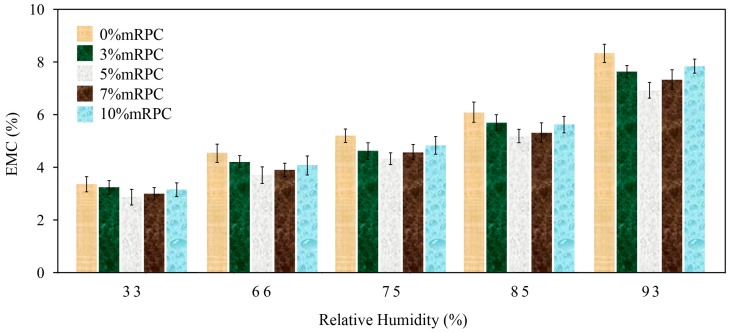
Comparison of the EMCs of the composites along the desorption direction.

Figure 2 and Figure 3 show the equilibrium moisture contents (EMCs) of the mRPC/HDPE/wood fiber composites when they reached the adsorption and desorption equilibrium, respectively, under different relative humidity conditions. The comparison of Figure 2 and Figure 3 suggests that adding the mRPC improved the moisture adsorption behavior of the HDPE/wood fiber composites. Under the same humidity conditions, the EMC reached through adsorption was lower than that reached through desorption, indicating the presence of an adsorption hysteresis phenomenon [31]. When the mass percentage of mRPC was increased, the EMC of the composite decreased.

**Figure 4:**
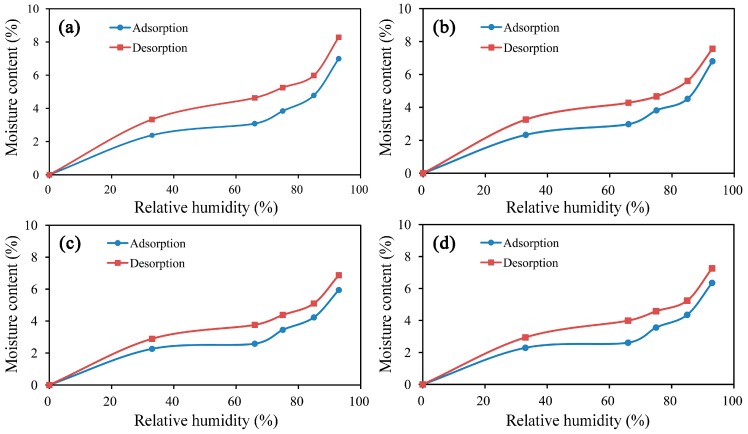

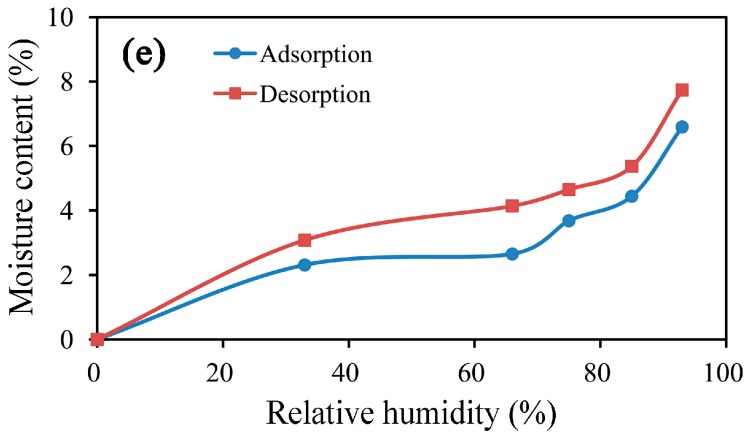
Comparative analysis of the adsorption and desorption behavior of the prepared composites containing different mass percentages of mRPC: (**a**) 0% mRPC; (**b**) 3% mRPC; (**c**) 5% mRPC; (**d**) 7% mRPC; (**e**) 10% mRPC.

The water adsorption isotherm obtained for the mRPC/HDPE/wood fiber composites resembles a sigmoid shaped curve, as shown in Figure 4. Compared with the isotherm obtained for the composite without the mRPC, the EMC along the adsorption direction was lower by 0.4%. The experimental results suggest that the mRPC caused the adsorption hysteresis of the composite. Compared with that of the composite without the mRPC, the EMC along the desorption direction decreased by 0.54%. The mRPC effectively decreased the water desorption of the WPCs. This result contributed in the reduced shrinkage rate and the enhanced dimensional stability of the composite. Therefore, the number of surface cracks occurred less, and the anti-aging performance of the composite was reinforced.

Table 2 and Table 3 revealed that the percentage of added mRPC effected the absorption and desorption of composite by Tukey test. Cross-comparison of the five groups of data shows that the third group (Sample 3) differs greatly from the other four groups. The results showed that adding the mRPC improved the moisture adsorption behavior of the HDPE/wood fiber composites. Under the same humidity conditions, the EMC of Sample 3 reached a lower value than Samples 1, 2, 4 and 5 in the adsorption and desorption, indicating the presence of an adsorption hysteresis phenomena. When the mass percentage of mRPC was increased to 5% (Sample 3), great influence occurred with respect to the absorption and desorption of the composite (Sig = 1); the EMC of the composite was optimal. The data of the Tukey test verified that the moisture adsorption characteristics of the composites were reinforced with mRPC.

### 3.3. Dynamic Thermal Mechanical Analysis

**Figure 5:**
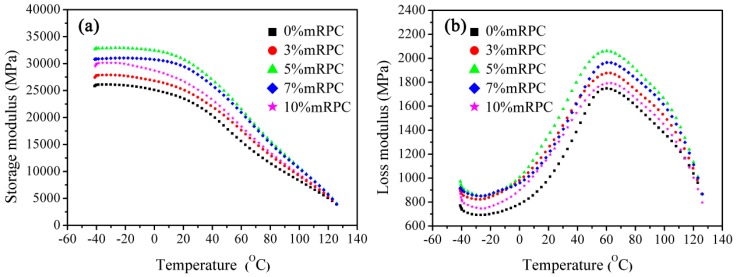
(**a**) Storage modulus and (**b**) loss modulus curves obtained for the mRPC/high-density polyethylene (HDPE)/wood fiber composites.

Figure 5 reveals the storage modulus and loss modulus curves of mRPC/HDPE/wood fiber composite samples. The storage modulus curve shows an overall downward trend with increasing temperature, which is in good agreement with the thermal mechanical performance of HDPE/wood powder/nanoclay blend composites that was observed by Deka et al. [9]. With increasing mRPC mass percentage, the storage modulus curve showed an obvious upward trend, as illustrated in Figure 5a. When the temperature was lower than 50 °C, the increase in amplitude of the storage modulus was relatively obvious; when the temperature was higher than 50 °C, the decrease in amplitude of the storage modulus became more obvious. In addition, the storage modulus curves of these samples tend to overlap. As the composite transformed from the glass state to the highly elastic state, the rigidity and storage modulus of the material decreased. The storage modulus of the sample with the mRPC was higher than that of pure HDPE, since the mRPC acts as a rigid structure in the composite. When the mass percentage of mRPC was 5%, the storage modulus of the composite was 3264 MPa, whereas the storage modulus decreased to 3003 MPa at a mass fraction of 10%. This suggests that an agglomeration occurs, which results in a decrease of the rigidity if the mRPC content is too high.

Figure 5b shows the loss modulus curve of the composite samples. In the curve, the obvious peak in the range from 50°–70° is the α-relaxation peak. The addition of the mRPC did not have a significant effect on the α-relaxation peak, whereas the loss modulus was more significantly enhanced. With increasing mRPC content, the mRPC showed a more network-like distribution in the matrix. The network-like distribution of the mRPC can greatly inhibit the flow of the composite melt, thereby increasing energy consumption. However, the loss modulus dropped by 16.8% when the mass percentage of mRPC was increased from 5%–10%, which indicates that an agglomeration of the mRPC destroyed the network-like distribution in the composite, thus causing the lower loss modulus.

At an mRPC mass percentage of 5%, the storage modulus and the loss modulus of the composite both reached an optimum. However, Kuo et al. reported an optimum of only 3 wt % clay [32], which indicates that the silane coupling agent can improve the interfacial compatibility between the mRPC and the WPC. Therefore, the optimum mRPC content in this study was found to be higher, and the dynamic thermal mechanical performance of the composite could be further enhanced.

**Figure 6:**
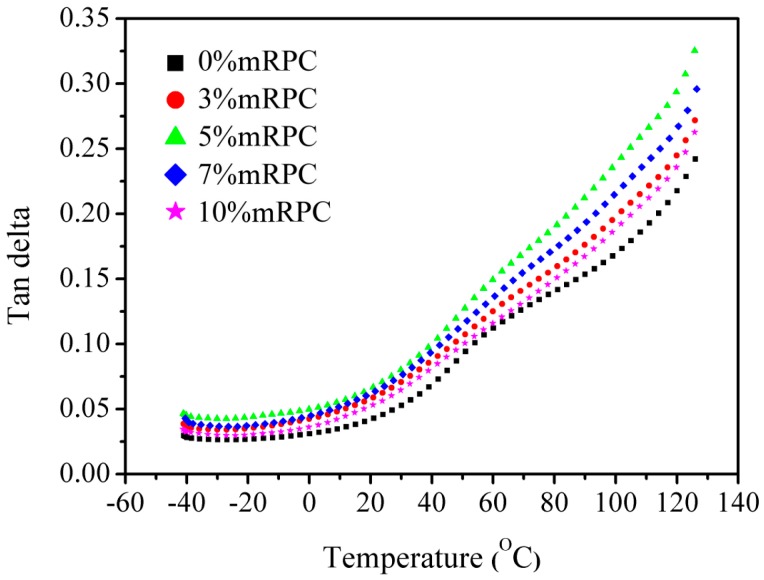
Loss-tangent curve obtained for the mRPC/HDPE/wood fiber composites.

Figure 6 shows the loss-tangent obtained for the modified RPC/HDPE/wood fiber composites. The results of the analysis suggest that, for temperatures below 70 °C, the addition of mRPC did not significantly change the tan *S* value, whereas the addition of the mRPC can increase the tan *S* when the temperature is higher than 70 °C. With increasing temperature, the tan *S* of the composite increased, indicating that the addition of the mRPC can enhance the viscosity of the composite. When the mass percentage of mRPC was 5%, the composite showed a more obvious elastic characteristic.

As mentioned above, the interfacial bonding between wood fiber and polymer matrix can also be enhanced using mRPC. Strong interactions between them at the interface tend to reduce the macromolecular mobility in the vicinity of the filler surface compared to that in the bulk matrix [33,34]. The A values for the composites at 40 °C are calculated by using Equation (1). The wood-plastic composites prepared without mRPC exhibited the highest *A* values of 0.813 (the weakest interfacial interaction); however, the wood-plastic composites prepared from mRPC by high temperature and pressurized treatment (145–165 °C, 10 MPa, 15 rpm/min) exhibited the strongest interactions (lowest *A* value: 0.495). This result demonstrated that high temperature and pressure strongly affected the interfacial bonding between wood fiber and plastic and also the properties of the prepared composites.

### 3.4. Thermogravimetry Analysis

**Figure 7:**
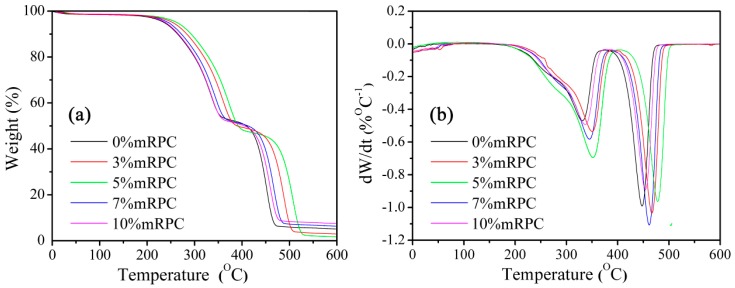
(**a**) TGA and (**b**) DTG curves of the mRPC/HDPE/wood fiber composites.

Figure 7 shows the weight-temperature curve obtained for the mRPC/HDPE/wood fiber composites, i.e., the TGA curve. The curve can be divided into two thermal degradation stages. The first thermal degradation stage comprises the temperature range from 310–380 °C, corresponding to the degradation of the wood fibers and the mRPC; the second thermal degradation stage comprises the temperature range from 460–510 °C, corresponding to the thermal degradation of HDPE. Both thermal degradation stages are associated with sharp increases of the slope of the TGA curve. The TGA curve indicates that the addition of different mass percentages of mRPC can cause different onset thermal degradation temperatures since the steric resistance of the mRPC influences the thermal flowability of the polymer chains and inhibits the reorganization of the polymer chains. When the mRPC mass percentage was 5%, the effect of the mRPC on the thermal degradation temperature of the composites was most obvious, which is consistent with the results published by Elshereafy et al., who reported that the addition of 10% organic montmorillonite clay can effectively increase the thermal degradation temperature of HDPE [18].

These two thermal degradation stages correspond to the sharp peaks on their derivative thermogravimetry analysis (DTG) curves. The DTG curves indicate that the addition of mRPC increases the thermal degradation peak temperature of the composite, resulting in a right-shift of the thermal degradation peak, and increases the temperature of the thermogravimetry with small amplitude. Therefore, adding the mRPC to the composite system can decrease the thermal degradation rate of the composite. The minimum thermal degradation rate of the composite was observed at an mRPC mass percentage of 5%.

### 3.5. Effect of the Mass Percentage of mRPC on the Mechanical Properties of the Composites after the UV-Accelerated Aging Process

**Figure 8:**
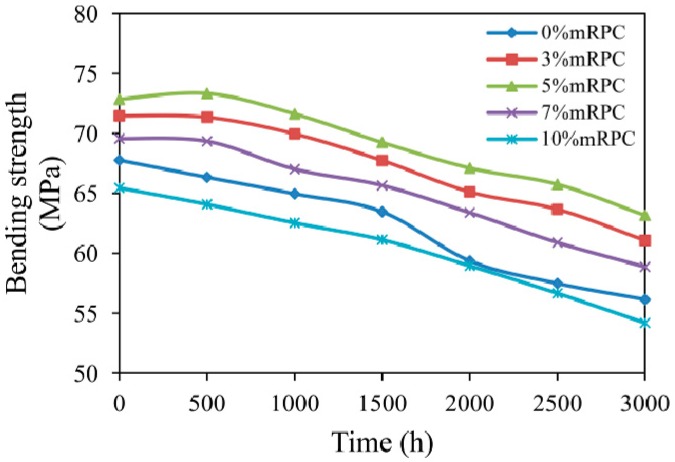
Effect of mRPC mass percentage and aging time on flexural strength.

Figure 8 shows the effect of mRPC mass percentage on the flexural strength of HDPE/wood fiber composites, which indicates that the addition of mRPC effectively enhances the flexural strength of the composite. Since during the modification process, the silane coupling agent was chemically adsorbed on the surface of the RPC, the mRPC remarkably improved dispersity in the composite and was uniformly dispersed on the surface of the material. This mRPC strengthened the interfacial bonding between the filler particles and the composite components, prevented a direct contact and the adsorption of UV light and enhanced the mechanical properties of the composite. Furthermore, the steam-silane complex modification can reduce the polarity of the wood fibers, increase their surface roughness and further improve the surface binding ability between the wood fibers and the plastic melt. When the mRPC mass percentage was 5%, the composite showed the best anti-aging performance.

For the composites containing mRPC mass percentages of 3% and 5%, the flexural strength of the composites showed a certain enhancement when the exposure time was in the range from 0–500 h. Apparently, under the action of the applied temperature, the rigid structure of the mRPC in the composite was strengthened, which led to a short enhancement of the anti-aging performance. However, when the UV-accelerated aging period was 1500 h, the flexural strength of the composite material with 5% mRPC decreased from 72.84 down to 69.24 MPa. This value was still the maximum value observed among the five groups of samples with different mRPC content. The flexural strength of the composite with 3% and 5% mRPC decreased by 5.21% and 4.94%, respectively. However, the flexural strength of these two samples was still higher than that of the composite material without mRPC before the aging process; when the UV-accelerated aging period was 3000 h, the flexural strength of the composite with 5% mRPC decreased to 63.17 MPa, which is still the maximum value observed among the five groups of samples. The flexural strength of the composites with 3%, 5%, 7% and 10% mRPC decreased by 14.53%, 13.28%, 15.37% and 17.02%, respectively, whereas the flexural strength of the composite material without mRPC decreased by 17.13%, which is the largest decrease.

**Figure 9:**
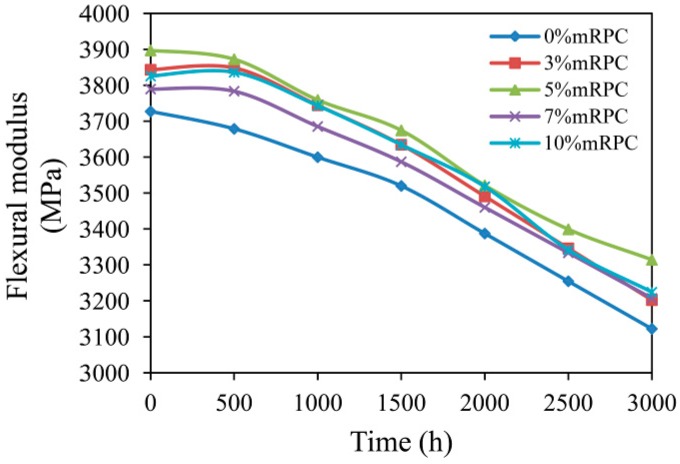
Effect of mRPC mass percentage and aging time on flexural modulus.

Figure 9 shows the effect of mRPC mass percentage and aging time on the flexural modulus. The addition of the mRPC effectively enhanced the flexural modulus of the composite. The UV light caused a certain surface breakage and cracks on the surface of the composite so that the flexural modulus of all samples tended to decrease. Before the aging test, the flexural strength of the composite without the mRPC was 3727.01 MPa, which is the lowest value among the five groups of samples. After an aging for 500 h, the flexural modulus of the composite without the mRPC decreased by 1.3%, whereas the flexural modulus of the other samples first increased and then decreased with increasing aging time after reaching a maximum. However, with increasing aging time, the flexural modulus decreased more rapidly; after an UV-accelerated aging for 1500 h, the flexural modulus of the composite with 5% mRPC decreased by 4.98%, but was still higher than the flexural modulus of the composite without mRPC before the aging; after an UV-accelerated aging for 3000 h, the flexural modulus of the composites with 3%, 5%, 7% and 10% mRPC decreased by 15.35%, 14.15%, 16.68% and 16.22%, respectively. However, the flexural modulus of the composite without mRPC decreased by 17.51%, which is the largest decrease.

**Figure 10:**
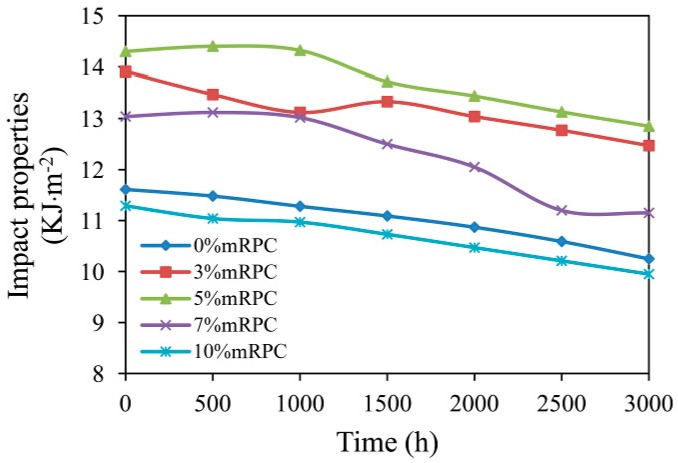
Effect of mRPC mass percentage and aging time on impact strength.

Figure 10 shows the effect of mRPC mass percentage on the impact strength. The addition of mRPC effectively enhanced the impact strength of the composite. Before the aging test, the impact strength of the composite without the mRPC was 11.61 MPa, which was the minimum value among the five groups of samples; after the samples were aged for 500 h, the impact strength of the WPCs increased to a certain extent. However, as the aging time was further increased, a loss in impact strength began to progressively occur; after an UV-accelerated aging for 1000 h, the impact strength of the composite with 5% mRPC was 14.32 MPa. The impact strength only slightly improved, since the crosslinking between the short-chain radicals is only a short-term phenomenon. However, as the aging time increased, the surface gaps of the composite became larger, and the impact strength finally decreased; after an UV-accelerated aging for 3000 h, the impact strengths of the composites with 3% and 5% mRPC were 12.47 and 12.85 MPa, which correspond to a decrease by 10.35% and 10.15%, respectively. At this time, the impact strength of the samples in these two groups was still higher than that of the composite without the mRPC before the aging test. Thus, the addition of mRPC can enhance the impact strength of WPCs after a UV-accelerated aging, and an mRPC mass percentage of 5% yielded the best results.

### 3.6. Effect of the mRPC Content on the Surface Color of the Composite after the UV-Accelerated Aging

**Figure 11:**
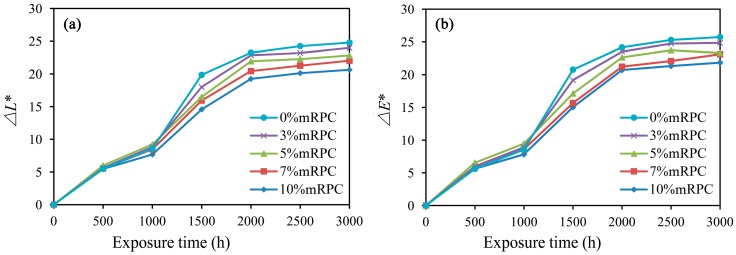
Effect of the duration of UV-accelerated aging treatment on the surface color of the composite (**a**) ΔL* (luminescence); (**b**) ΔE* (color).

The surface color of mRPC/HDPE/wood fiber composites varied with the duration the samples were exposed to the UV-accelerated aging, as shown in Figure 11. When mRPC was added to the composite, there are similar trends for the changes in luminescence (ΔL*) and color (ΔE*). With increasing aging time, the ΔL* and the ΔE* values increased, and the surface fading became more evident. Photodegradation and yellowing of WPCs have been observed in many studies, and hence, several hypotheses have been proposed to explain the mechanisms of the color changes [35]. Compared with the UV-accelerated aging performance of wood/PVC composites studied by Yang et al., the ΔL* and ΔE* values showed a similar change trend [25]. However, the wood powder/PVC composites showed a more serious surface-fading phenomenon, due to the lack of a reinforcing (due to no UV inhibition) treatment [7]. For an aging period between 0 and 1000 h, ΔL* and ΔE* both showed a smaller change; for an aging period between 1000 and 2000 h, ΔL* and ΔE* increased more significantly and almost linearly. For aging times higher than 2000 h, the ΔE* value still increased, but the overall trend became smoother. After 3000 h of UV-accelerated aging, the ΔL* and ΔE* values of the composite with 10% mRPC were 20.636 and 21.845, corresponding to the lowest difference in surface color. These characterization results indicate that, under the action of UV light, the chromophores of the mRPC became briefly active, and then, the chromophores generated a colorless substance by oxidation, resulting in the surface fading of the composite. The photo-degradation reaction caused the breakdown of the HDPE chains in the non-crystalline regions and a higher brightness on the sample surface. However, by adding the mRPC, the photo-degradation reaction was effectively prevented, which helped to preserve the surface color.

### 3.7. Effect of the mRPC Content on the Microstructure and Surface Morphology of the Composite after the UV-Accelerated Aging

**Figure 12:**
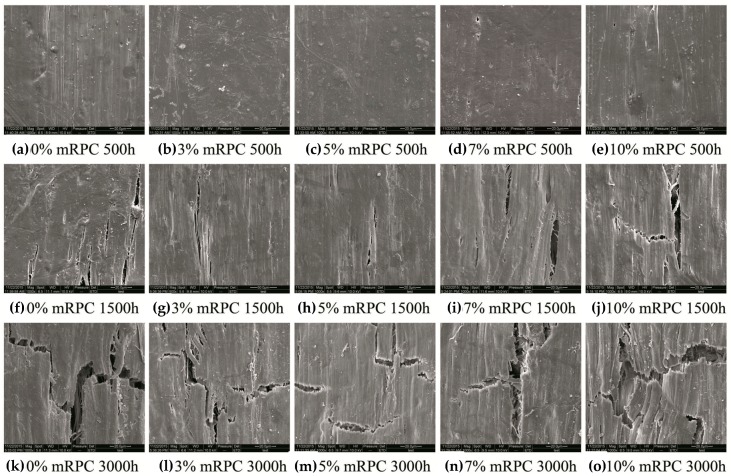
Surface morphology of the composite samples with different mRPC content after the UV-accelerated aging (magnification 1000×).

Figure 12 shows TEM micrographs revealing the surface morphology of the mRPC/HDPE/wood fiber composites after the UV-accelerated aging. As shown in Figure 12a–e, after 500 h of UV-accelerated aging, no obvious cracks were found on the surfaces of the five types of samples. The composite samples with 3% mRPC (b), and the composite samples with 5% mRPC (c) exhibited the most regular and smooth surfaces. For these two surfaces, the HDPE matrix enveloped and protected the wood fibers well, and the surfaces of the composite are smooth. The mRPC is uniformly dispersed inside and on the surface of the composite, which further enhances the anti-aging performance of the composites.

After being subjected to 1500 h of UV-accelerated aging test, the surface of the composite without mRPC exhibited the largest cracks as shown in Figure 12f–j. The composite samples with 3% mRPC (g) and the composite with 5% mRPC (h) had much less cracks than the composite without mRPC (f). The composite sample with 10% mRPC had larger surface cracks than those with 3%, 5% and 7% mRPC. The mRPC to a certain degree blocks the UV light, so that the UV light cannot act directly on the wood fibers. Instead, it is transformed into heat energy, which then dissipates. This mechanism resulted in a decrease of the degradation rate of the HDPE, slowed the degradation of the composite and led to the generation of some tiny cracks on the surface of the sample, which enhanced the anti-aging performance of the composite.

As revealed in Figure 12k–o, after 3000 h of UV-accelerated aging, the cracks on the surface of the composite sample without the mRPC (k) further grew and the number of cracks increased. The same results were also reported by Matuana et al. [36]. However, the cracks on the surface of the composite sample with 5% mRPC (m) were relatively smaller. This composite showed the best anti-aging performance, which was attributed to the fact that the diameter of the mRPC particles was between 100 and 180 nm, so that the particles could effectively fill the gaps between the wood fibers and the HDPE matrix and inhibit the degradation of the interface between fibers. Therefore, the interface is less prone to cracking. The composite with 10% mRPC (o) was most seriously affected by surface cracks since the mRPC mass percentage was too high, which led to an agglomeration of the mRPC particles. The agglomerated mRPC particles cannot effectively block UV light, so that the interface between the wood fibers and the HDPE was exposed. The exposed area was degraded under the action of the UV light so that larger surface cracks occurred.

## 4. Conclusions

Within the scope of this study, the main conclusion is that the silane, NH_2_CH_2_CH_2_CH_2_Si(OC_2_H_5_)_3_, is an effective coupling agent for modifying red pottery clay to enhance HDPE/wood fiber composites. The subordinate conclusions under the main conclusion are:

1. With increasing mRPC mass percentage, the moisture adsorption isothermal curve resembled an “S”-shaped upward curve.
2. The equilibrium moisture content of the composite along the adsorption and the desorption direction both increased, whereas the equilibrium moisture content along the adsorption direction was less than that along desorption direction, resulting in a moisture hysteresis.
3. The mRPC particles act as a rigid structure in the composite and significantly improve the rigidity, so it can be applied to enhance the elastic properties of the composite at elevated temperatures. The composite with 5% mRPC showed the best performance. The storage modulus was 3264 MPa, and the loss modulus increased by 16.8% due to the addition of the mRPC.
4. Dynamic thermal mechanical analysis indicates the mRPC showed a more network-like distribution in the matrix. At an mRPC mass percentage of 5%, the storage modulus and the loss modulus of the composite both reached an optimum. The addition of the mRPC can enhance the viscosity of the composite. The composite showed a more obvious elastic characteristic.
5. Thermal gravimetric analysis indicates that the inhibiting effect of the mRPC can influence the thermal flowability of the polymer chains and prevent their reorganization. Thus, the addition of the mRPC can increase the thermal degradation peak temperature and effectively decrease the thermal degradation rate. The composite samples with 5% mRPC showed the highest enhancement on the thermal degradation temperature.
6. The flexural strength, the flexural modulus and the impact strength continuously decreased with the aging time. After an aging for 3000 h, the composite with 5% mRPC showed the smallest reduction in mechanical performance, with a flexural strength, flexural modulus and impact strength of 63.17 MPa, 3284.17 MPa and 12.85 MPa, respectively.
7. The luminescence (ΔL*) and color (ΔE*) values both increased with the aging time; the maximum increase in amplitude was observed for aging times between 0 and l000 h, whereas the ΔL* and ΔE* values gradually increased after l000 h, while at the same time, the overall curve tended to become smoother. The composite with 5% mRPC showed the lowest degree of fading.

## Figures and Tables

**Table 1:** Composition of the red pottery clay used in this study.

Mineral Composition	wt %
MgO	6.57
Al_2_O_3_	16.93
SiO_2_	68.23
Fe_2_O_3_	8.27

**Table 2:** Tukey test of the EMCs of the composites along the moisture adsorption direction.

Sample	MeanDiff	SEM	q Value	Prob	Alpha	Sig
1,3	−0.522	1.05567	0.69974	0.98695	0.05	1
2,3	−0.398	1.05567	0.53352	0.99533	0.05	1
4,3	0.140	1.05567	0.18767	0.99992	0.05	1
5,3	0.242	1.05567	0.32440	0.99933	0.05	1

Note: Mean Difference (MeanDiff); Standard Error of Mean (SEM); False Discovery Rate (q value); Probit (Prob); Alpha (Alpha); Significance (Sig).

**Table 3:** Tukey test of the EMCs of the composites along the desorption direction.

Sample	MeanDiff	SEM	q Value	Prob	Alpha	Sig
1,3	−0.894	1.05567	1.19764	0.91253	0.05	1
2,3	−0.476	1.05567	0.63767	0.99079	0.05	1
4,3	0.202	1.05567	0.27061	0.99967	0.05	1
5,3	0.396	1.05567	0.53050	0.99544	0.05	1
